# Maison De Santé in New York

**Published:** 1860-06

**Authors:** 


— *A Maison de Santé* has recently been opened in New York, the announcement of which, we believe, will be received with pleasure by the profession. The arrangements are such as will be highly beneficial to the interests of any physician who may have a patient requiring careful attendance, or who may need the comforts of a home. It is under the professional management of Dr. Henry Schweig, and is located in Second Avenue, near Ninth Street. We shall give an account of this establishment in our next number.

